# Circle of Willis variations in migraine patients with ischemic stroke

**DOI:** 10.1002/brb3.1223

**Published:** 2019-02-16

**Authors:** Arend M. Hamming, Marianne A. A. van Walderveen, Inge A. Mulder, Irene C. van der Schaaf, L. Jaap Kappelle, Birgitta K. Velthuis, Michel D. Ferrari, Gisela M. Terwindt, Marieke C. Visser, Wouter Schonewille, Ale Algra, Marieke J. H. Wermer, the Dutch acute Stroke Study (DUST) investigators

**Affiliations:** ^1^ Department of Neurology Leiden University Medical Center Leiden The Netherlands; ^2^ Department of Radiology Leiden University Medical Center Leiden The Netherlands; ^3^ Department of Radiology University Medical Center Utrecht Utrecht The Netherlands; ^4^ Department of Neurology University Medical Center Utrecht Utrecht The Netherlands; ^5^ Department of Neurology VU Medical Center Amsterdam The Netherlands; ^6^ Department of Neurology Antonius Hospital Nieuwegein The Netherlands; ^7^ Department of Clinical Epidemiology Leiden University Medical Center Leiden The Netherlands; ^8^ The Julius Center University Medical Center Utrecht Utrecht The Netherlands; ^9^ Department of Neurology and Neurosurgery, Brain Center Rudolf Magnus University Medical Center Utrecht Utrecht The Netherlands

**Keywords:** circle of Willis, computed tomography, humans, migraine, neuroimaging, strokes

## Abstract

**Objectives:**

Migraine is a risk factor for stroke, which might be explained by a higher prevalence in anatomical variants in the circle of Willis (CoW). Here, we compared the presence of CoW variants in patients with stroke with and without migraine.

**Materials and Methods:**

Participants were recruited from the prospective Dutch acute Stroke Study. All participants underwent CT angiography on admission. Lifetime migraine history was assessed with a screening questionnaire and confirmed by an interview based on International Classification of Headache Disorders criteria. The CoW was assessed for incompleteness/hypoplasia (any segment <1 mm), for anterior cerebral artery asymmetry (difference > 1/3), and for posterior communicating artery (Pcom) dominance (Pcom–P1 difference > 1/3). Odds ratios with adjustments for age and sex (aOR) were calculated with logistic regression.

**Results:**

We included 646 participants with stroke, of whom 52 had a history of migraine. Of these, 45 (87%) had an incomplete or hypoplastic CoW versus 506 (85%) of the 594 participants without migraine (aOR: 1.47; 95% CI: 0.63–3.44). There were no differences between participants with and without migraine in variations of the anterior or posterior CoW, anterior cerebral artery asymmetry (aOR: 0.86; 95% CI: 0.43–1.74), or Pcom dominance (aOR: 0.64; 95% CI: 0.32–1.30). There were no differences in CoW variations between migraine patients with or without aura.

**Conclusion:**

We found no significant difference in the completeness of the CoW in acute stroke patients with migraine compared to those without.

## 1 INTRODUCTION

The circle of Willis (CoW) is an important structure for collateral cerebral blood flow. Anatomical variations of the CoW are common in the general population. CoW variants may be congenital but can also be acquired when patients get older (Zaninovich, Ramey, Walter, & Dumont, 2017). Some (Bugnicourt et al., 2009; Cavestro et al., 2011; Cucchiara et al., 2013; Henry et al., 2015), but not all (Schoonman, van Oosterhout, Ferrari, & van der Grond, 2010; Ezzatian‐Ahar et al., 2014; Ikeda et al., 2017), studies report a higher frequency of incomplete CoW in migraine patients compared with controls in particular for the posterior circulation and in patients with migraine with aura.

Migraine with aura is associated with a twofold risk of ischemic stroke (Spector et al., 2010). In addition, migraine with aura is strongly associated with subclinical infarctions in the posterior circulation (Kruit, van Buchem, Launer, Terwindt, & Ferrari, 2010). Variation in the anatomy of the CoW, notably in the posterior circulation, might contribute to the increased risk of stroke in migraine patients like in patients with cardiovascular disease (Hartkamp, van Der Grond, van Everdingen, Hillen, & Mali, 1999; Vrselja, Brkic, Mrdenovic, Radic, & Curic, 2014; van Seeters et al., 2015). An incomplete CoW might hamper collateral blood flow through the CoW in case of an ischemic event and might also affect cerebral perfusion, possibly facilitating spreading depolarizations (SDs, Cucchiara et al., 2013). Spreading depolarizations are the electrophysiological correlate for migraine aura, and most commonly affect the visual cortex in the posterior circulation territory (Bugnicourt et al., 2009; Hadjikhani et al., 2001). Higher susceptibility to SDs decreases the threshold for cerebral ischemia (Dohmen et al., 2008; Dreier, 2011; Eikermann‐Haerter et al., 2012). Thus, the combination of SDs and CoW variations might be a risk factor for stroke in migraine.

The relationship between migraine and CoW variants has thus far only been investigated in population or out‐patient‐based migraine cohorts (Bugnicourt et al., 2009; Cavestro et al., 2011; Cucchiara et al., 2013; Ikeda et al., 2017). One would expect, however, that if there is a real relationship between variation in the CoW and migraine, this should be more pronounced in patients with stroke. In the present study, we tested this hypothesis.

## 2 MATERIALS AND METHODS

### 2.1 Patients

Participants were included from the Dutch acute Stroke Study (DUST), a large prospective multicenter cohort study performed between May 2009 and August 2013 in the Netherlands (ClinicalTrials.gov NCT00880113) (van Seeters et al., 2014). The aim of DUST was to investigate the value of CT perfusion (CTP) and CT angiography (CTA) for predicting outcome after ischemic stroke (van Seeters et al., 2014). Inclusion criteria were age ≥ 18 years, onset of stroke symptoms < 9 hr, and NIHSS  ≥ 2 or ≥1 if intravenous thrombolysis was indicated. Exclusion criteria were other diagnosis than ischemic stroke on CT scan, known renal failure, or known contrast allergy. Between February 2011 and August 2013, 10 of the 14 participating hospitals (University Medical Center Utrecht (UMCU), Alysis, Catharina Ziekenhuis, St Radboud Nijmegen, Gelre Hospital Apeldoorn, Leiden University Medical Center (LUMC), Medisch Centrum Haaglanden (MCH), St Elisabeth Ziekenhuis, VU Medical Center (VUmc), and St Antonius Ziekenhuis) included patients in the migraine side‐study.

Demographic data, medical history, cardiovascular risk factors, and NIHSS score on admission were prospectively recorded. Stroke territory was assessed by the treating physician with access to clinical and radiological data. DUST was approved by the medical ethical committee of the participating hospitals. A written informed consent was obtained from all patients for use of their data.

### 2.2 Migraine assessment

Lifetime migraine history was assessed in a uniform way by the DUST research nurses with the short 5‐item migraine in stroke screener (MISS). The MISS questionnaire was validated in a previous study and has a high negative predictive value (0.99) but a moderate positive predictive value (0.80) in patients with stroke (van der Willik, Pelzer, Algra, Terwindt, & Wermer, 2016). Therefore, all patients who answered positively to any of the questions were contacted by a research assistant, trained by a migraine neurologist (G.M.T), for an extensive migraine interview to verify the migraine diagnosis. The migraine interview was based on the International Classification of Headache Disorders (ICHD)‐II criteria (Headache Classification Subcommittee of the International Headache Society, 2004) which are comparable to the recently updated ICHD‐III beta criteria (Headache Classification Committee of the International Headache Society, 2013). Migraine diagnoses were divided into two subtypes: (a) migraine with aura and (b) migraine without aura. Patients who had both migraine attacks with and without aura were included in the migraine with aura group. Patients who fulfilled the criteria for migraine without aura but had probable or possible aura symptoms were classified as migraine without aura. Patients who refused to participate in the telephone interview or were lost to follow‐up were excluded from the analysis.

### 2.3 Assessment of anatomical variations in the circle of Willis

All patients underwent noncontrast CT (NCCT), CTP, and CTA on admission with standardized scan protocols between centers. The scan parameters for the NCCT were 120 kVp, 300 mAs, and 1 mm reconstructed slice thickness. For CTA, 60–80 mL of contrast agent (300 mg I/mL) was injected into the antecubital vein (18‐gauge needle) at a rate of 6 mL/s followed by a 40 mL saline flush at a rate of 6 mL/s. The scan parameters for the CTA were 120 kVp, 150 mAs, and 1 mm reconstructed slice thickness. Radiologic parameters were assessed by one of three neuroradiologists with at least 5 years of experience in stroke imaging (van Seeters et al., 2015).

Our primary endpoint was incompleteness of the CoW. Segments of the CoW were considered normal if they had a diameter of ≥1 mm. Segments <1 mm were classified as hypoplastic or invisible. The anterior CoW was classified as incomplete if the anterior communicating artery or one of the A1 segment(s) of the anterior cerebral artery were hypoplastic or invisible. The posterior CoW was classified as one‐sided incomplete if one of the posterior communicating arteries (Pcom) or P1 segments of the posterior cerebral artery was hypoplastic or invisible. If a hypoplastic or invisible Pcom or P1 segment was present on both sides, the posterior CoW was classified as two‐sided incomplete.

A1 asymmetry was considered present if the diameter of the left and right A1 segments differed by more than one‐third. The Pcom was considered dominant if the Pcom diameter exceeded the ipsilateral P1 diameter by more than one‐third. Additional variants of the CoW were noted, such as a median artery corpus callosi (MACC, three A2 segments) and an azygos anterior cerebral artery. Patients with incomplete radiological data on the CoW anatomy were excluded. For a subgroup analysis excluding patients with large vessel disease, we excluded all patients with a stenosis >70% or occlusion in a large vessel (common and internal carotid, basilar an vertebral arteries) as visible on CT angiography.

### 2.4 Data analysis

Anatomical variations of the CoW were compared between stroke patients with and without a history of migraine. Within the patients with migraine, we compared CoW variations in patients with and without aura. Odds ratios (OR) and 95% confidence intervals (CI) were calculated with univariable and multivariable (aOR, adjusted for age and sex) logistic regression analyses. Data were analyzed with IBM SPSS Statistics for Windows, Version 20.0.

## 3 RESULTS

### 3.1 Patients

In total, 866 DUST patients were included in the participating hospitals during the period of collecting the MISS migraine questionnaire. Of those, 707 (82%) participants filled the questionnaire. In total, 32 were lost to follow‐up, 25 refused to participate in the telephone interview, and in four, the radiological data on the CoW were incomplete for technical reasons. We therefore included 646 patients in our study; 52 with a history of migraine and 594 without migraine. Of the 52 patients with migraine, 29 (56%) had migraine with aura and 23 (44%) had migraine without aura.

Stroke patients with migraine were in general younger, more often female, and had less often hypertension compared with stroke patients without migraine (Table 1). Migraine with aura patients more often had ischemia in the posterior circulation compared to migraine without aura patients and patients without migraine. Stroke subtypes were scored according to the etiological TOAST classification (as introduced in the Trial of Org 10172 in Acute Stroke Treatment) (Adams et al., 1993). Stroke subtypes were comparable between the groups except that small vessel disease was more often found to be the cause of stroke in migraine patients with aura.

**Table 1:** Baseline characteristics of the 646 participants

	No migraine *N* = 594	Migraine *N* = 52	MA *N* = 29	MO *N* = 23	*p* value (migraine vs. no migraine)
Age (mean, ±SD)	67 (±13)	60 (±11)	58 (±8)	64 (±13)	0.0003
Women	221 (37%)	29 (56%)	12 (41%)	17 (74%)	0.01
Smoker (*N* = 615)	168 (30%)	23 (45%)	13 (45%)	10 (45%)	0.03
Alcohol use (*N* = 496)	263 (62%)	26 (72%)	14 (70%)	12 (75%)	0.46
NIHSS (median, *N* = 642)^a^	5 (7)	5 (9)	5 (9)	4 (7)	0.36
Stroke/TIA history (*N* = 641)	138 (23%)	14 (27%)	7 (24%)	7 (30%)	0.57
Hypertension (*N* = 639)	284 (48%)	20 (38%)	10 (34%)	10 (43%)	0.29
Stroke territory					
ACA (*N* = 596)	23 (4%)	1 (2%)	0 (0%)	1 (6%)	0.56
ACM (*N* = 595)	426 (77%)	29 (67%)	17 (65%)	12 (71%)	0.15
Posterior territory (*N* = 616)	98 (17%)	11 (23%)	8 (30%)	3 (14%)	0.32
Stroke type (*N* = 416)					
Large vessel disease	169 (44%)	13 (42%)	7 (47%)	6 (38%)	0.99
Cardiac embolus	96 (25%)	8 (26%)	3 (20%)	5 (31%)	
Small vessel disease	78 (20%)	7 (23%)	4 (27%)	3 (19%)	
Dissection	22 (6%)	2 (6%)	1 (7%)	1 (6%)	
Other	20 (5%)	1 (3%)	0 (0%)	1 (6%)	

*Notes*

MA: migraine with aura; MO: migraine without aura; NIHSS: National Institutes of Health Stroke Scale; ACA: anterior cerebral artery; ACM: middle cerebral artery. Smokers only include current smokers. Alcohol use is any degree of alcohol consumption. Hypertension refers to a history of hypertension prior to the stroke. Stroke territory was scored by the treating physician, with access to radiological data. Posterior stroke territory includes both the posterior cerebral artery (PCA) and basilar and vertebral artery territories. Stroke type is according to TOAST classification, with dissections specified from other causes. (*N*) = The number of patients for the particular variable in case there are missing data. *p* values, for migraine versus no migraine, are calculated with an independent samples Student's *t* test for age, a Mann–Whitney *U* test for NIHSS, and a chi‐square test for the other variables. Characteristics with *p* values <0.05 do not have to be confounders whereas characteristics with *p* values >0.05 may still be confounders. We considered age and sex to be confounders and adjusted for them in our analyses.

a parentheses: interquartile range.

### 3.2 Variants of the circle of Willis

Of the 52 migraineurs, 45 (87%) had an incomplete CoW versus 506 (85%) of the 594 participants without migraine (OR 1.12; 95% CI 0.49–2.56) (Table 2). After adjustment for age and sex, the aOR was 1.47 (95% CI 0.63–3.44). There were also no differences between the two groups when the anterior and posterior CoW were analyzed separately. Asymmetry of the A1 segment of the anterior cerebral artery (aOR 0.86; 95% CI 0.43–1.74) and dominance of the Pcom (aOR 0.64; 95% CI 0.32–1.30) were also not different in migraineurs. Migraine with aura patients more often had an incomplete anterior CoW compared with participants without migraine (aOR 3.22; 95% CO 1.21–8.59). There were no differences in posterior of total CoW incompleteness between migraine with aura and participants without migraine.

**Table 2:** CoW variants in stroke patients with and without migraine

	Migraine	MA	No migraine	Migraine versus no migraine		MA versus no migraine	
	(*N* = 52)	(*N* = 29)	(*N* = 594)	OR (95% CI)	aOR (95% CI)	OR (95% CI)	aOR (95% CI)
CoW incomplete	45 (87%)	23 (79%)	506 (85%)	1.12 (0.49–2.56)	1.47 (0.63–3.44)	0.67 (0.26–1.68)	0.88 (0.34–2.29)
Anterior incomplete	8 (15%)	6 (21%)	61 (10%)	1.59 (0.71–3.53)	2.06 (0.90–4.73)	2.28 (0.89–5.82)	3.22 (1.21–8.59)
Posterior incomplete							
One‐sided	16 (31%)	7 (24%)	183 (31%)	1.00 (0.54–1.84)	0.95 (0.51–1.78)	0.71 (0.30–1.70)	0.70 (0.29–1.67)
Two‐sided	28 (54%)	15 (52%)	318 (54%)	1.01 (0.57–1.79)	1.22 (0.68–2.20)	0.93 (0.44–1.96)	1.12 (0.52–2.39)
A1 asymmetry (*N* = 645)	11 (21%)	6 (21%)	156 (26%)	0.75 (0.38–1.50)	0.86 (0.43–1.74)	0.73 (0.29–1.83)	0.82 (0.32–2.07)
Pcom dominance	11 (21%)	6 (21%)	173 (29%)	0.65 (0.33–1.30)	0.64 (0.32–1.30)	0.63 (0.25–1.59)	0.67 (0.26–1.72)

*Notes*

OR: odds ratio (with the 95% confidence interval); aOR: odds ratio adjusted for age and sex (with the 95% confidence interval); MA: migraine with aura; CoW: circle of Willis; Pcom: posterior communicating artery.

(*N*) = The number of patients for the particular variable in case there are missing data.

In total, 23 (79%) of migraine with aura patients had an incomplete CoW versus 22 (96%) migraine without aura patients (aOR 0.14; 95% CI 0.01–1.58) (Table 3). There was no difference in A1 asymmetry or Pcom dominance between the two subtypes of migraine. Additional variants (most commonly the MACC) were found in 4% of all patients and were not more frequent in migraineurs.

**Table 3:** CoW variants in migraine patients with and without aura

	MA	MO	MA versus MO	
	(*N* = 29)	(*N* = 23)	OR (95% CI)	aOR (95% CI)
CoW incomplete	23 (79%)	22 (96%)	0.17 (0.02–1.57)	0.14 (0.01–1.58)
Anterior CoW incomplete	6 (21%)	2 (9%)	2.74 (0.50–15.09)	4.18 (0.58–30.00)
Posterior CoW incomplete				
One‐sided	7 (24%)	9 (39%)	0.49 (0.15–1.63)	0.45 (0.12–1.67)
Two‐sided	15 (52%)	13 (57%)	0.82 (0.27–2.48)	0.85 (0.24–2.97)
A1 asymmetry	6 (21%)	5 (22%)	0.94 (0.25–3.58)	0.93 (0.21–4.17)
Pcom dominance	6 (21%)	5 (22%)	0.94 (0.25–3.58)	1.55 (0.35–6.81)

*Notes*

OR: odds ratio (with the 95% confidence interval); aOR: odds ratio adjusted for age and sex (with the 95% confidence interval); MA: migraine with aura; MO: migraine without aura; CoW: circle of Willis; Pcom: posterior communicating artery.

(*N*) = The number of patients for the particular variable in case there are missing data***.***

In patients with ischemia in the posterior circulation, incompleteness of the posterior CoW was not more common in migraineurs than in patients without migraine (aOR 0.81; 95%CI 0.16–4.14).

In a subgroup analysis excluding patients with large vessel disease, no significant differences were found between participants with versus no migraine, nor in participants with migraine with aura versus no migraine in unadjusted nor adjusted odds ratios, comparing total, anterior and posterior completeness, A1 asymmetry, nor Pcom dominance (Tables S2b and S3b).

## 4 DISCUSSION

In our ischemic stroke cohort, anatomical variations of the CoW were equally common in patients with or without a history of migraine and in migraineurs with or without aura.

Several studies have reported an increased prevalence of CoW variants in people with migraine in comparison to the controls, in particular in migraineurs with aura and in the posterior circulation (Bugnicourt et al., 2009; Cavestro et al., 2011; Cucchiara et al., 2013; Henry et al., 2015). However, in most studies, the frequency of CoW variants in the control group was less than 50% which is considerably lower than expected from population based studies (El‐Barhoun, Gledhill, & Pitman, 2009; Kapoor, Singh, & Dewan, 2008; Krabbe‐Hartkamp et al., 1998; Li et al., 2011; Riggs & Rupp, 1963) and the 85% we found (Table 4).

**Table 4:** Studies reporting CoW anatomy in migraine populations

	Cucchiara et al. (2013)	Cavestro et al. (2011)	Bugnicourt et al. (2010)	Schoonman et al. (2010)	Ikeda et al. (2017)	Ezzatian‐Ahar et al. (2014)^b^	This study
Design	CC	CC?	CC	CC	CC?	CC	CC
Data collection	Pro	Pro	Pro	Retro	Retro	Retro	Pro
Controls	Age and sex matched Pt	Pt with no headache	Pt with other neurol diseases	Non‐migraine Pt	Unknown	Healthy controls	Non‐migraine stroke Pt
N	170	429	124	44	173	84	646
Non‐migraine	53	159	77	12	100	37	594
Migraine (% MA)	117 (48%)	204 (32%)	47 (51%)	32 (27%)	73 (42%)	48 (0%)	52 (56%)
Mean age (SD)	33.3 (6.6)	44.8 (14.9)	38.7 (14.8)	42.9 (9.3)	33.2 (8.9)	28 (MO), 25 (C)	66 (13)
Women	132 (78%)	314 (73%)	86 (69%)	25 (78%)	122 (71%)	84 (100%)	250 (39%)
Imaging	MR	MR	MR	MR	MR	MR	CT
Incomplete posterior CoW^c^							
Migraine	71 (61%)^a^	82 (30%)^a^	23 (49%)^a^	16 (50%)	28 (38%)^a^	n/a	44 (85%)
MA	36 (64%)^a^	24 (36%)^a^	14 (61%)	n/a	6 (19%)^a^	n/a	22 (76%)
MO	35 (57%)	58 (28%)^a^	9 (38%)	n/a	22 (52%)^a^	20 (43%)	22 (96%)
Control	22 (41%)	26 (16%)	14 (18%)	8 (67%)	55 (55%)	15 (41%)	501 (84%)
Anterior CoW							
Migraine	31 (26%)^a^	26 (10%)^a^	3 (6%)	3 (9%)	n/a	n/a	8 (15%)
MA	18 (32%)^a^	6 (9%)	2 (9%)	n/a	n/a	n/a	6 (21%)
MO	13 (21%)	20 (10%)^a^	1 (4%)	n/a	n/a	n/a	2 (9%)
Control	7 (13%)	8 (5%)	4 (5%)	2 (17%)	n/a	n/a	61 (10%)

Percentages are percentage incomplete posterior CoW as detected on MR or CT angiogram.

Pt: patients; C: controls; M: Median; Pro: prospective; Retro: retrospective; CC: case–control study; C: cohort study; MA: migraine with aura; MO: migraine without aura.

a Statistically significant difference compared with controls. Note that in this table, in contrast to Table 2, both MA and MO patients were compared with controls rather than with each other. Also, note that Ikeda found a lower prevalence of incomplete CoW compared with controls.

b Ezzatian‐Ahar et al. did not discriminate between anterior and posterior incompleteness of the CoW.

c A part of this table is derived from the paper of Cucchiara et al.

Some of the variation in frequency of CoW anomalies among previous studies may have been due to differences in scoring criteria. We used a cut‐off of 1 mm for incompleteness because it is known from previous autopsy and flow model studies that segments below this diameter significantly compromise blood flow (Alpers, Berry, & Paddison, 1959; Cassot et al., 1995; Waaijer et al., 2007; Schomer et al., 1994). For the same reason, we chose a one‐third difference for A1 asymmetry and Pcom dominance (Cassot et al., 1995; Waaijer et al., 2007). Most other MRI studies used a cut‐off of 0.8 mm. Also, the age and sex distribution of the study populations varied between studies. Our stroke cohort consisted of relatively old persons which may account for the relatively high proportion of CoW variants as CoW variation is more common in elderly (Krabbe‐Hartkamp et al., 1998). Our results suggest more frequent incompleteness of the anterior circle in migraine patients with aura compared to stroke patients without migraine. We feel this finding should be interpreted with caution because it was not reported in previous studies, it was based on only 6 migraine patients with aura and a pathophysiological explanation for the difference is lacking.

This is the first study that investigated the association between CoW variants and migraine in a stroke population. The strengths of our study are the prospective data collection, the large number of participants, the verified migraine diagnosis, and the detailed investigation of the CoW by trained neuroradiologists. However, our study also has limitations. Not all DUST patients answered the MISS migraine questionnaire and 8% of the patients could not be contacted for a telephone interview. Because of the etiological nature of the study, we tried to avoid misclassification bias and only included patients with a negative questionnaire or a verified migraine diagnosis. Therefore, the exact prevalence of migraine in our stroke population cannot be derived from our study. In addition, we cannot exclude that some patients who reported on the MISS screener not to have a migraine history might not have accurately recalled their migraine symptoms when asked about it many years later. Furthermore, we cannot exclude that CoW morphology changed because of the stroke, given the plastic nature of CoW anatomy (Chuang et al., 2009). However, since all patients in our study had a stroke we feel that it is unlikely that this affected the internal validity of our study. In addition, all patients were scanned in the first hours after onset excluding chronic adaptations of the CoW after stroke. Also, chronic changes to the CoW may have occurred because of atherosclerotic changes related to aging (Rutgers, Klijn, Kappelle, van Huffelen, & van der Grond, 2000). To address this problem, we corrected for age. In addition, we performed a subgroup analyses in which we excluded patients with large vessel stenosis or occlusions. In this subgroup analyses, our results stayed essentially the same. An ultimate future study would focus on the longitudinal relation between migraine symptoms and CoW morphology.”

While there is radiological and genetic evidence for a relationship between CoW variants and stroke, causality is debated (Hartkamp et al., 1999; de Monye et al., 2008; Hoksbergen et al., 2003; Mawet, Kurth, & Ayata, 2015). In a prospective follow‐up study in patients with atherosclerotic disease, an incomplete (<0.8 mm or absent segment) anterior and posterior CoW was related to future anterior circulation stroke (van Seeters et al., 2015). An incomplete CoW might decrease the possibilities for collateral blood flow and might cause shear induced platelet aggregation and possibly facilitates SDs (van Raamt, Mali, van Laar, & van der Graaf, 2006; Borgdorff & Tangelder, 2014; Russell & Olesen, 1996). However, other studies also suggested that the CoW mainly functions as a pressure absorber (Vrselja et al., 2014). In case the CoW is a redundancy mechanism where a secondary route takes over perfusion of the brain when the primary route is blocked, an incomplete circle may contribute to the chance of developing ischemia (Schomer et al., 1994). It can be hypothesized that patients with an incomplete posterior CoW with migraine are more susceptible for ischemia in the posterior territories. However, in our patients with posterior ischemia, there were no differences in completeness of posterior CoW in migraineurs compared with patients without migraine. We did not investigate the influence of pial and leptomeningeal collaterals. Future studies are needed to assess their role in the vascularization of the posterior circulation in patients with migraine and to assess the longitudinal relationship between migraine and CoW morphology.

## CONFLICT OF INTEREST

There are no conflicts of interest.

## FUNDING INFORMATION

Dr. Wermer was supported by a personal grant from the Netherlands Organization for Scientific Research (ZonMW Veni grant), the Dutch Heart Foundation (2011T055), and the Dutch Brain Foundation (F2014(1)‐22). The DUST study was supported by a grant from the Dutch Heart Foundation (2008T034) and the NutsOhra Foundation (0903‐012).
