# Supplementary material for: Circle of Willis variations in migraine patients with ischemic stroke
Source: Brain Behav. 2019 Feb 16;9(3):e01223. doi: 10.1002/brb3.1223 (PMC6422794; doi:10.1002/brb3.1223)
Supplement: Supplementary file 1 [file BRB3-9-e01223-s001.docx]

***Table 2b*** CoW-variants in stroke patients with and without migraine; subgroup without large vessel disease.

|  | Migraine | MA | no Migraine | Migraine vs. no Migraine | | MA vs. no Migraine | |
| --- | --- | --- | --- | --- | --- | --- | --- |
|  | (N=47) | (N=25) | (N=491) | OR (95% CI) | aOR (95% CI) | OR (95% CI) | aOR (95% CI) |
| CoW incomplete | 41 (87%) | 20 (80%) | 416 (85%) | 1.23 (0.51-3.00) | 1.66 (0.66-4.19) | 0.72 (0.26-1.98) | 0.99 (0.35-2.82) |
| Anterior incomplete | 7 (15%) | 5 (20%) | 53 (11%) | 1.45 (0.62-3.39) | 1.81 (0.75-4.39) | 2.066 (0.74-5.73) | 2.81 (0.97-8.14) |
| Posterior incomplete |  |  |  |  |  |  |  |
| - One-sided | 15 (32%) | 6 (24%) | 146 (30%) | 1.12 (0.58-2.11) | 1.07 (0.55-2.07) | 0.75 (0.29-1.91) | 0.74 (0.29-1.91) |
| - Two-sided | 25 (53%) | 13 (52%) | 266 (54%) | 0.96 (0.53-1.75) | 1.16 (0.62-2.16) | 0.92 (0.41-2.05) | 1.11 (0.49-2.54) |
|  |  |  |  |  |  |  |  |
| A1 asymmetry (N=645) | 10 (21%) | 5 (20%) | 120 (24%) | 0.83 (0.40-1.73) | 0.93 (0.44-1.95) | 0.77 (0.28-2.10) | 0.83 (0.30-2.29) |
| Pcom dominance | 10 (21%) | 5 (20%) | 145 (30%) | 0.64 (0.31-1.33) | 0.62 (0.29-1.31) | 0.60 (0.22-1.62) | 0.59 (0.21-1.65) |

***Legend***

OR = odds ratio (with the 95% confidence interval), aOR = odds ratio adjusted for age and sex (with the 95% confidence interval).

MA = migraine with aura

CoW = Circle of Willis

Pcom = posterior communicating artery

(N) = The number of patients for the particular variable in case there are missing data

***Table 3b*** CoW-variants in migraine patients with and without aura; subgroup without large vessel disease.

|  | MA | MO | MA vs. MO | |
| --- | --- | --- | --- | --- |
|  | (N=25) | (N=22) | OR (95% CI) | aOR (95% CI) |
| CoW incomplete | 20 (80%) | 21 (95%) | 0.29 (0.02-1.78) | 0.14 (0.01-1.59) |
| Anterior CoW incomplete | 5 (20%) | 2 (9%) | 2.50 (0.43-14.43) | 4.00 (0.55-29.34) |
| Posterior CoW incomplete |  |  |  |  |
| - One-sided | 6 (24%) | 9 (41%) | 0.46 (0.13-1.59) | 0.39 (0.10-1.52 |
| - Two-sided | 13 (52%) | 12 (55%) | 0.90 (0.29-2.85) | 0.93 (0.26-3.34) |
|  |  |  |  |  |
| A1 asymmetry | 5 (20%) | 5 (23%) | 0.85 (0.21-3.44) | 0.88 (0.19-4.10) |
| Pcom dominance | 5 (20%) | 5 (23%) | 0.85 (0.21-3.44) | 1.31 (0.29-5.98) |

***Legend***

OR = odds ratio (with the 95% confidence interval), aOR = odds ratio adjusted for age and sex (with the 95% confidence interval).

MA = migraine with aura, MO = migraine without aura

CoW = Circle of Willis

Pcom = posterior communicating artery

(N )= The number of patients for the particular variable in case there are missing data
